# DNase1 reverses peribronchial inflammation in murine explants incubated with activated neutrophils

**DOI:** 10.3389/fimmu.2026.1883328

**Published:** 2026-08-31

**Authors:** Hong Zhang, Jasmin Knopf, Julia Elrod, Michaela Klinke, Michael Boettcher, Richard Martel

**Affiliations:** 1 Department of Pediatric Surgery, University Medical Center Mannheim, Medical Faculty Mannheim, Heidelberg University, Mannheim, Germany; 2 European Center for Angioscience (ECAS), Medical Faculty Mannheim, Heidelberg University, Mannheim, Germany; 3 Mannheim Institute for Innate Immunoscience (MI3), Medical Faculty Mannheim, Heidelberg University, Mannheim, Germany

**Keywords:** neutrophil extracellular traps, DNase1, inflammation, Ccl5, lung innate immunity, precision-cut lung explants

## Abstract

**Introduction:**

Neutrophil extracellular traps (NETs) are chromatin-based structures released by activated neutrophils that are associated with microvascular thrombosis and pulmonary inflammation. These are adverse effects that are relevant in ECMO-associated lung injury. Although enzymatic NET degradation by DNase1 represents a potential anti-inflammatory strategy, its anti-inflammatory effects in the lung tissue and on vascular endothelial growth factor (VEGF) signalling have received limited investigation. We used a tissue-architecture–preserving murine lung explant model to characterise inflammation following exposure to activated neutrophils and to determine whether DNase1 attenuates this response without compromising VEGF expression.

**Methods:**

Murine lung tissue in an explant culture was exposed to phorbol 12-myristate 13-acetate–activated human neutrophils. DNase1 was used to prevent NET-mediated effects. Inflammatory and angiogenic responses were assessed using multiplex proximity extension assay and quantitative immunofluorescence of peribronchiolar regions of interest.

**Results:**

In whole-explant lysates, the pre-specified primary analyte C-C motif chemokine ligand 5 (Ccl5) was significantly increased following exposure to activated neutrophils (p = 0.015); among exploratory analytes, C-C motif chemokine ligand 3 remained significant after false-discovery-rate correction (Ccl3 p = 0.005, q = 0.014), while C-X-C motif chemokine ligand 9 was nominally elevated (Cxcl9 p = 0.041, q = 0.061). Immunofluorescence confirmed increased Ccl5, in the peribronchiolar regions (p = 0.002, q = 0.004), which was significantly attenuated by DNase1 (p = 0.019, q = 0.038). Vascular endothelial growth factor expression was not significantly altered by neutrophil exposure or DNase1 treatment (p ≥ 0.29).

**Discussion:**

NET-associated inflammation in murine lung explants is detected within peribronchiolar regions of interest and is attenuated by DNase1-mediated degradation of extracellular DNA without compromising VEGF expression. These findings provide a rationale for further evaluating DNase1 as a candidate adjunct anti-inflammatory strategy in ECMO-associated lung injury, where the preservation of VEGF expression alongside NET inhibition is potentially attractive given the bleeding risks of conventional anticoagulation. Clinical applicability requires confirmation in flow-based ex vivo systems, *in vivo* models, and human ECMO studies.

## Introduction

Extracorporeal membrane oxygenation (ECMO) is a life-saving cardiopulmonary support but is associated with a high incidence of thrombotic and haemorrhagic complications, particularly in neonates, where reported rates reach 18 % and 17 % respectively (1–3). A central driver of these is complications the sterile inflammatory response triggered when blood contacts the artificial circuit surface (4), which activates neutrophils and induces the release of neutrophil extracellular traps (NETs) (5). NETs are web-like chromatin structures contributing to immunothrombosis, microvascular occlusion, and acute lung injury when accumulated in excess (6). Activated neutrophils release NETs, leading to platelet activation and inflammatory signalling, contributing to acute lung injury during ECMO (7). Therefore, NET modulation is clinically relevant in ECMO and may represent a strategy to reduce inflammatory burden and adverse effects of anticoagulation.

Conversely, NET-associated proteases can positively modulate proangiogenic mediators such as vascular endothelial growth factor (VEGF) (8), as described in macrovascular models (9) and degradation of NETs has been associated with impaired vascular regeneration in selected ischemia models (10, 11).

DNase1, an endogenous endonuclease, degrades extracellular DNA, the structural backbone of NETs, without directly interfering with canonical coagulation pathways. Experimental studies have demonstrated that DNase1 limits NET-mediated microvascular occlusion and attenuates ischaemia-associated tissue injury *in vivo* (12–15), suggesting its potential as a modulator of NET-driven inflammation.

We hypothesised that NET deposition affects lung inflammation that can be pharmacologically uncoupled, thereby providing a mechanistic rationale for NET-targeted intervention. Thus, a murine lung explant model was established to (i) interrogate whether experimentally induced NETs-formation is associated with inflammation in the lung parenchyma, (ii) determine whether enzymatic NET disruption by DNase1 reverses this inflammatory program, and (iii) assess whether such intervention spares VEGF-expression.

## Methods

### Study design

**Figure 1:**
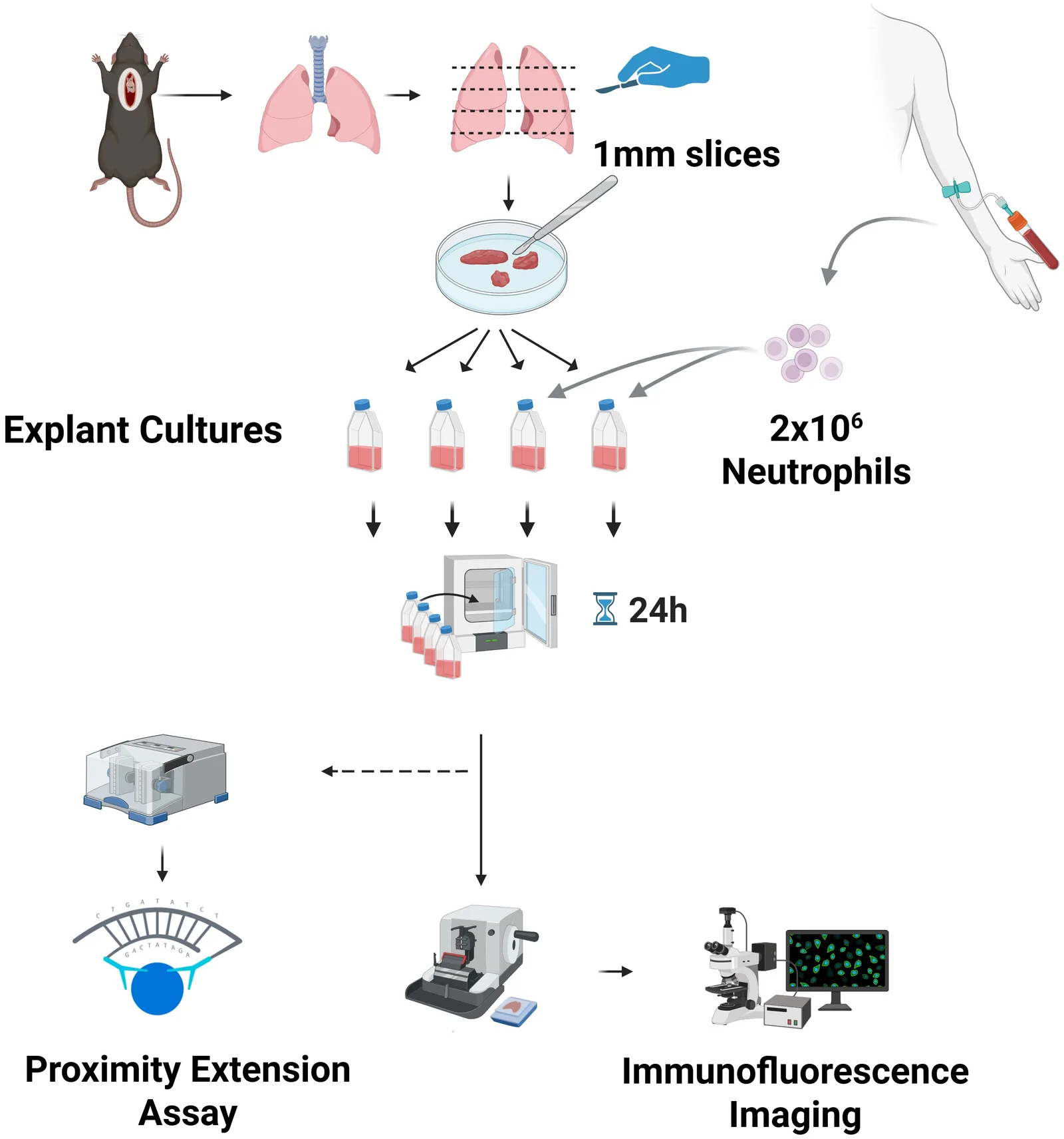
Murine lung slices subjected for subsequent experimental series, for 1) NETs exposure and 2) anti-NET intervention. Pairs of 1mm lung tissue slices from murine lungs were incubated for 24h under four different expositions. The first series comprised Medium only control (MOC), phorbol 12-myristate 13-acetate (PMA) only, non-stimulated Neutrophils (Neutro) and PMA-stimulated Neutrophils (Neutro+PMA) and was explored by a multiplex protein assay and further by immunofluorescence imaging. A further series to test anti-NET intervention included MOC, Neutro+PMA, Neutro+PMA with DNase (Neutro+PMA+DNase) and DNase only. Neutrophils were derived from peripheral human blood. Created in BioRender. Kinderchirurgie, L. (2026).

Pairs of lung tissue slices were prepared from five animals in two experimental series for incubation under four different experimental conditions, respectively (**Figure 1**). In a first series (N = 5), the model of exposure to phorbol 12-myristate 13-acetate (PMA)-activated neutrophils (Neutro+PMA) was tested against medium only control (MOC), PMA only and non-stimulated Neutrophils (Neutro). Inflammation and angiogenetic signalling were explored by proximity extension assay (PEA) in the first half of the tissue pairs. Another experimental series with N = 5 animals was performed to examine anti-NET intervention by the following conditions: MOC, Neutro+PMA, DNase1 intervention (Neutro+PMA+DNase) and DNase1 only.

### Explant culture and neutrophil preparation

All experiments were carried out after approval by the local animal welfare office (approval number I-20/22). Institutional guidelines for animal welfare and experimental conduct were followed. All procedures complied with Directive 2010/63/EU and were reported in accordance with the ARRIVE 2.0 guidelines. Lungs from BALB/c mice (Janvier labs, France) were harvested immediately after euthanasia by deep isoflurane anaesthesia followed by decapitation. 1 mm-thick tissue slices were prepared utilising a microtome blade. Two slices respectively were maintained as an explant culture (16) per 25 ml cell culture flask with filter cap (Sarstedt, Nuembrecht, Germany) containing 4ml DMEM/F12 (Gibco, Thermo Fisher Scientific, Waltham, MA, USA) and RPMI-1640 medium (Gibco), (1:1) with addition of 1mM MgCl_2_ (Carl Roth, Karlsruhe, Germany). Peripheral venous blood (45 mL) was collected from healthy adult donors by venepuncture with EDTA-blood collection systems (Sarstedt, Nuembrecht, Germany) following written informed consent in accordance with institutional ethical guidelines (vote number 2023-587). Neutrophils were isolated using the MACSxpress^®^ Whole Blood Neutrophil Isolation Kit, human (Miltenyi Biotec, Bergisch Gladbach, Germany) according to the manufacturer’s instructions, with incubation on a MACSmix™ Tube Rotator and magnetic separation to yield untouched neutrophils (17), the pellet was diluted in 5 ml Phosphate Buffered Saline. Neutrophil concentration was counted in the LUNA-FL™ Dual Fluorescence Cell Counter (Logos Biosystems, South Korea), stained with Acridine Orange/Propidium Iodide Staining Solution (Cat# E23001, Logos Biosystems, South Korea). For Neutro+PMA, 100 nM phorbol 12-myristate 13-acetate (Sigma-Aldrich, Saint Louis MO, USA) was added to induce NET formation (18). In each cell culture flask of the Neutro, Neutro+PMA and Neutro+PMA+DNase group, 2×10^6^ Neutrophils were suspended to the medium and incubated together with the lung tissue slices for 24 hours.

### Multiplex protein assay

Initial cytokine profiling and quantification of VEGF expression was performed using a proximity extension assay (PEA) to quantify multiple proteins simultaneously (Target 96 mouse exploratory, olink, Uppsala, Sweden) (19, 20). The Target 96 mouse exploratory assay is designed to detect murine marker proteins with highest sensitivity. The respective tissue slices were homogenised in Tissue Protein Extraction Reagent (T-PER) lysis buffer (Thermo Fisher Scientific, Rockford IL, United States) supplemented with 20 µl per 1 mg tissue buffer protease inhibitor (phenylmethanesulfonyl fluoride solution, Sigma-Aldrich, Saint Louis MO, USA) using a bead-based tissue homogeniser (Qiagen Retsch TissueLyser, Qiagen, Venlo, Netherlands). Lysates were centrifuged (10,000 rpm, 5 min, 4 °C), and supernatants were collected and stored at −80 °C. Protein concentrations were determined using a Bradford assay (Bio-Rad Laboratories Inc., Hercules CA, USA) with BSA standards (Sigma-Aldrich, Saint Louis MO, USA) prepared in the same lysis buffer. Samples were normalised to 1 mg/ml total protein, and appropriate dilutions (undiluted, 1:4, and 1:16) were prepared prior to the multiplex protein analysis by olink. The results were provided by olink as arbitrary units on a log2 scale. Only inflammatory mediators and angiogenic markers were considered for statistical testing.

### Immunostaining of tissue sections

Paraffin-embedded tissue samples were sectioned at a thickness of 3 µm using a microtome (RM2235, Leica, Nussloch, Germany). Sections were deparaffinised at 60 °C for 60 minutes and immersed in xylene three times for 5 minutes each. Slides were subsequently rehydrated by graded ethanol (100% ×2, 96%, 90%, 80%, 70%). The MaxBlock™ Autofluorescence Reducing Kit (MaxVision Biosciences, MVB-MB-L, Hamburg, Germany) was used according to the instructions. Antigen retrieval was performed in 10 mM sodium citrate buffer (pH 6.0) at 90 °C for 20 min, followed by cooling at room temperature for 60–90 minutes. Tissues were permeabilised with 0.2% Triton X-100 in PBS, blocked with 10% donkey or goat serum and incubated with primary antibodies at 4 °C overnight: Rabbit monoclonal anti-human/mouse/rat citrullinated histone H3 (anti-CitH3) 1:100 (Abcam, Amsterdam, Netherlands; catalogue number: ab219406), rabbit polyclonal anti-human/mouse/rat C-C motif chemokine ligand 5 (anti-Ccl5) 1:75 (Bio-Techne Ltd, Abingdon, UK, catalogue number: NB120-10394), rabbit polyclonal anti-human/mouse vascular endothelial growth factor (anti-VEGF) 1:150 (Sigma-Aldrich, Saint Louis MO, USA; catalogue number: SAB5700716). Secondary antibodies Donkey anti-goat Alexa Fluor 488, Donkey anti-rabbit Alexa Fluor 647, Goat anti-rabbit Alexa Fluor 488, Goat anti-rat Alexa Fluor 647 (Thermo Fisher Scientific, Rockford IL, United States, catalogue numbers: A32814, A32795, A11008, A21247) were incubated at room temperature in the dark, with a dilution of 1:200–1000 for 1 hour. DNA was stained using DAPI for 5 minutes at a dilution of 1:500 (Sigma-Aldrich, Saint Louis MO, USA; catalogue number: D9542-1MG). All samples were mounted with Dako Fluorescent Mounting Medium (Agilent, Santa Clara, CA 95051, United States, catalogue number: S3023).

### Image analysis

Immunofluorescence images were acquired as single optical planes using a Keyence BZ-X810 fluorescence microscope (Keyence, Osaka, Japan) equipped with 10x and 40× objective lenses (numerical apertures 0.45, 0.95). Images were captured using identical acquisition settings across all samples of each animal, including exposure time (blue 1/5 - 1/3 ms, green 1.5–3 ms, red 1/2.3 - 1/1.5 ms), gain (6 dB), and illumination intensity 100%. The field of view was 362 µm × 272 µm, and no background subtraction or post-acquisition brightness or contrast adjustments were applied during image acquisition.

Digital image analysis was performed using Fiji/ImageJ version 1.53c (National Institute of Health, USA). Entire images were analysed without cropping. Thresholding was applied uniformly across image stacks for each staining condition, while original images remained unaltered throughout the analysis. One image in the MOC of the first experimental series showing detector saturation due to overexposure was excluded from quantitative analysis. Images were taken by a blinded observer at 10x magnification for analysis of CitH3 and at 40x magnification for the analysis of Ccl5 and VEGF centring the largest bronchioles of the individual slice. Mean intensity ratios of red (Ccl5) and green (VEGF) against blue (DAPI) of entire images were analysed for group differences. To further control for bias, automated analysis was conducted on complete image stacks by the Measure-Stack tool using identical threshold settings across all samples. Colocalised expression (of CitH3 and MPO) was identified by the colocalization threshold tool in ImageJ. Constant intensity was used for colocalised pixels.

### Statistics

Statistical analysis was performed using SAS 9.4 (SAS Institute Inc., Cary, North Carolina USA) and GraphPad Prism 11 (GraphPad Software, Boston, Massachusetts, USA). Results are presented as arithmetic means ± standard deviation. Olink data on the log2 scale was transformed to linear values before statistical analysis. Normalised group means and individual values were obtained by dividing by the MOC mean. Because paired tissue slices originating from the same animal were distributed across experimental conditions, group effects were assessed in a mixed-effects model with experimental condition as a fixed effect and animal as a random (blocking) factor, so that the animal rather than the individual tissue slice constituted the experimental unit. The comparison between the model group (Neutro+PMA) and MOC and the DNase1-intervention group was specified *a priori* as the primary contrast and was analysed by pairwise comparison of least-squares means. The proinflammatory multiplex analytes (Ccl3, Ccl5, Cxcl9, Ccl20) were treated as exploratory endpoints and adjusted for multiple comparisons within this exploratory panel using the Benjamini-Hochberg procedure to control the false discovery rate (FDR), while vascular endothelial growth factor (VEGF) was analysed separately. For the immunofluorescence marker sets, the Benjamini-Hochberg procedure was likewise applied. Both unadjusted and FDR-adjusted significance-values (q) are reported, and nominally significant exploratory findings are interpreted as hypothesis-generating. Statistical significance was defined as p <no><</no> 0.05 (two-sided). Given the limited sample size (N = 5 slices per group), all analyses are interpreted as exploratory and hypothesis-generating.

## Results

**Figure 2:**
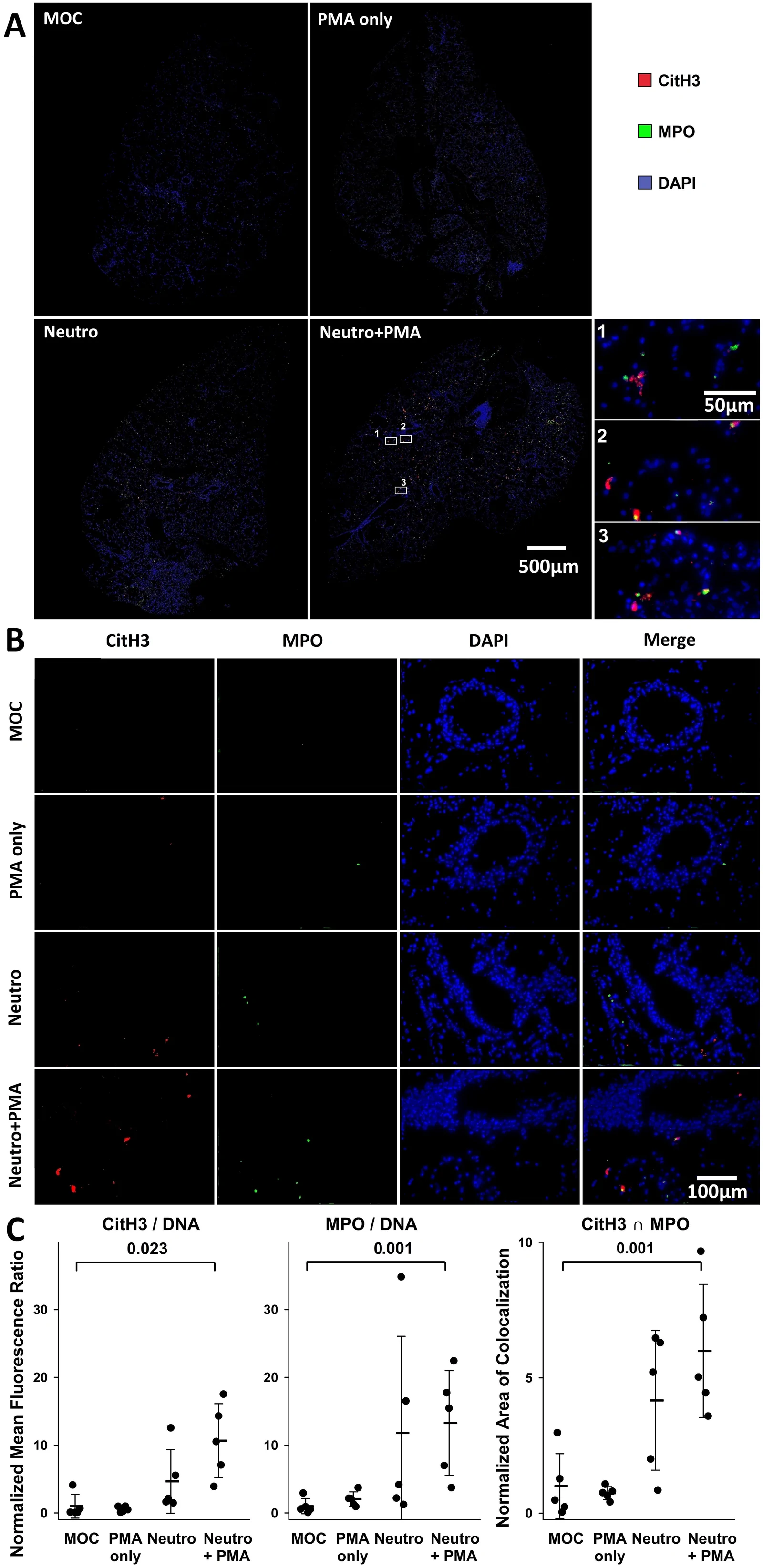
NETs form in murine lung explants following 24h exposition to activated neutrophils. **(A)** Immunofluorescence staining for citrullinated histone H3 (CitH3) and myeloperoxidase (MPO) identifies chromatin decondensation and extracellular DNA release as hallmarks of neutrophil extracellular trap (NET) formation. This effect was observed throughout the wholemount explants. Details highlight diffuse chromatin structures (NETs) extending beyond intact nuclei. **(B)** Immunofluorescence of peribronchiolar regions demonstrates a significant increase after Neutro+PMA in peribronchial CitH3 and MPO signal intensity normalised to total DAPI-positive nuclei. **(C)** This was evaluated by automated image analysis. Consistently, the area of colocalised CitH3 and MPO expression was significantly larger after Neutro+PMA indicating enhanced NET formation after exposure to PMA-activated neutrophils (n = 5 slices per condition from five animals, graphs show mean ± standard deviation, values above brackets indicate Holm-adjusted significance values). CitH3, citrullinated histone H3; DAPI, 4′,6-Diamidin-2-phenylindol-dihydrochlorid,2-(4-Amidinophenyl)-6-indolcarbamidin; MOC, medium only control; MPO, myeloperoxidase; PMA, phorbol 12-myristate 13-acetate; Neutro, neutrophils; Neutro+PMA, PMA-activated neutrophils.

After 24 hours of exposure to PMA-activated neutrophils (Neutro+PMA), the NET-markers citrullinated histone H3 (CitH3) and myeloperoxidase (MPO) were robustly detected within the explant tissue. Diffuse chromatin structures extending beyond intact nuclei, consistent with extracellular DNA fibres representing characteristic patterns of NETosis, were detected by immunofluorescence. These structures were found dispersed throughout the lung explant tissue after Neutro+PMA to a markedly greater extent than Neutrophils only (Neutro), PMA only or medium only (MOC) (**Figure 2A**). CitH3 and MPO expression was increased compared to medium-only control, PMA-only control, and unstimulated neutrophils (**Figure 2B**) as confirmed as statistically significant by quantitative image analysis (CitH3 p = 0.023, q = 0.023, n = 5; MPO p = 0.0008, q = 0.001, n = 5; **Figure 2C**). The area of colocalised CitH3 and MPO expression was significantly higher after Neutro+PMA compared to MOC (p = 0.0002, q = 0.001 n = 5; **Figure 2C**).

**Figure 3:**
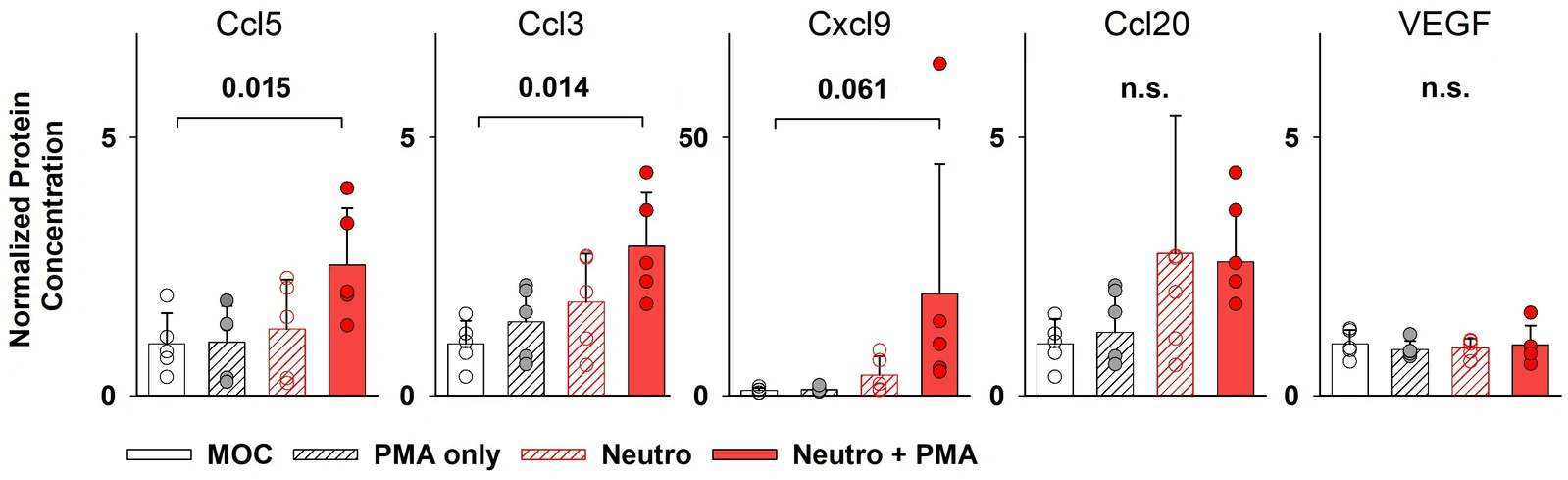
Whole explant lysates show elevated cytokines after NETs exposure. Multiplex protein exploration revealed significant elevation of the cytokines Cxcl9, Ccl3, Ccl5. VEGF presented equal levels in all experimental conditions (n = 5 lysates per condition from five animals, graphs show mean ± standard deviation, values above brackets indicate Holm-adjusted significance values). Cxcl9, C-X-C motif chemokine ligand 9; Ccl3, C-C motif chemokine ligand 3; Ccl5, C-C motif chemokine ligand 5; Ccl20, C-C motif chemokine ligand 20; MOC, medium only control; MPO, myeloperoxidase; PMA, phorbol 12-myristate 13-acetate; Neutro, neutrophils; Neutro+PMA, PMA-activated neutrophils.

Multiplex protein exploration in tissue lysates of whole explants showed that the pre-specified primary analyte, C-C motif chemokine ligand 5 (Ccl5), was significantly elevated following exposure to Neutro+PMA (p = 0.015, n = 5). Among the exploratory proinflammatory analytes, C-C motif chemokine ligand 3 (Ccl3) remained significant after control of the false discovery rate (p = 0.005, q = 0.014, n = 5), whereas C-X-C motif chemokine ligand 9 (Cxcl9) was nominally elevated (p = 0.041, n = 5) but did not retain significance after FDR correction (q = 0.061, n = 5), and C-C motif chemokine ligand 20 (Ccl20) was not significantly altered (p = 0.127, q = 0.127, n = 5). Vascular endothelial growth factor (VEGF) was unchanged (p = 0.92, n = 5; **Figure 3**).

**Figure 4:**
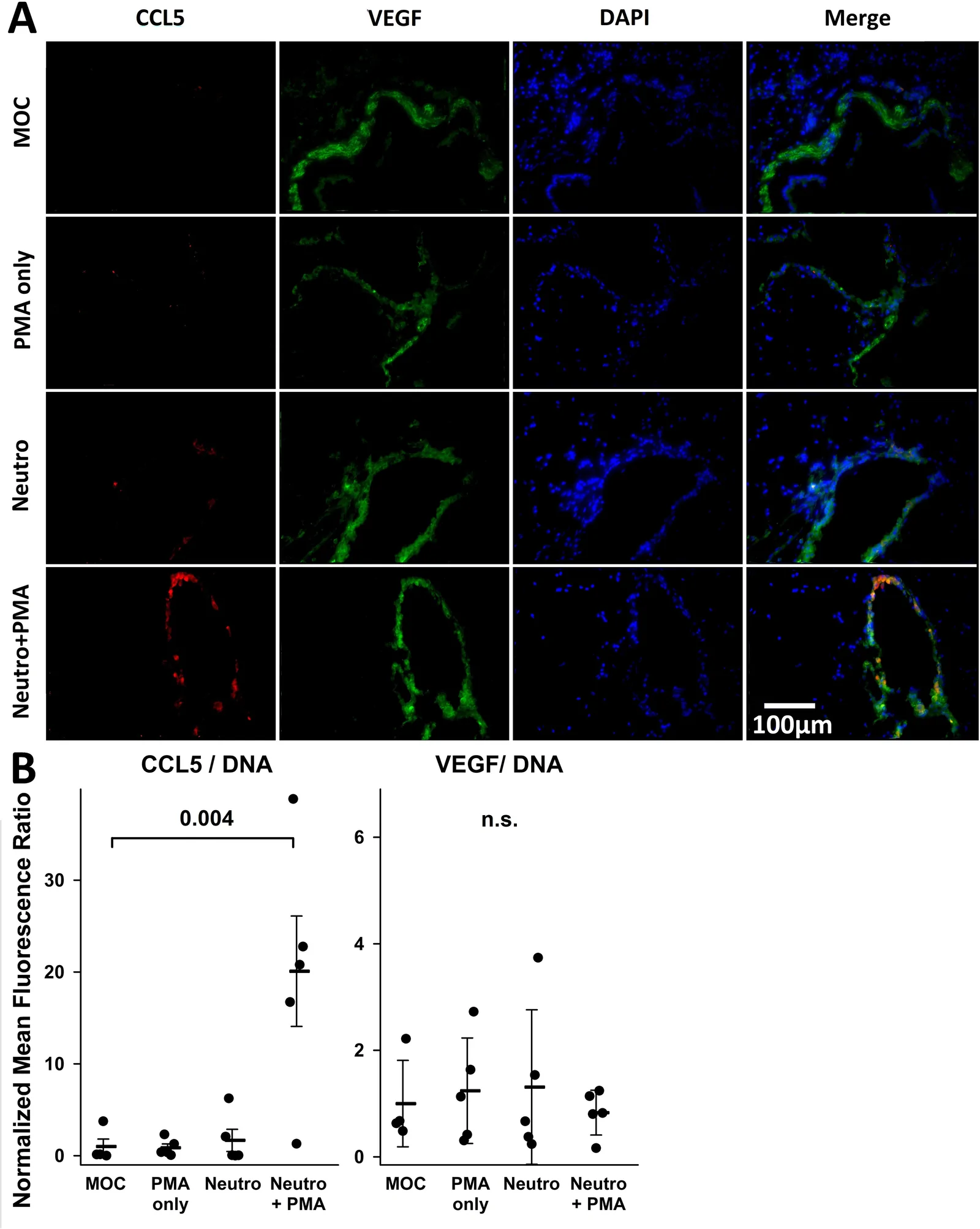
NET exposure induces localised inflammatory signalling in murine lung explants without altering VEGF expression. **(A)** Immunofluorescence demonstrates increased Ccl5 expression in peribronchiolar regions following exposure to activated neutrophils. **(B)** Automated quantitative image analysis confirms significantly elevated Ccl5 intensity normalised to total DAPI signal compared with control conditions. In contrast, VEGF expression remained unchanged across experimental groups (n = 5 slices per condition from five animals – one slice was excluded in the MOC group (MOC: n = 4), graphs show mean ± standard deviation, values above brackets indicate Holm-adjusted significance values). Ccl5, C-C motif chemokine ligand 5; DAPI, 4′,6-Diamidin-2-phenylindol-dihydrochlorid,2-(4-Amidinophenyl)-6-indolcarbamidin; DNase1, deoxyribonucleic acid degrading endonuclease – 1; MOC, medium only control; PMA, phorbol 12-myristate 13-acetate; Neutro, neutrophils; Neutro+PMA, PMA-activated neutrophils; VEGF, vascular endothelial growth factor.

Consistent with these findings, immunofluorescence imaging demonstrated increased Ccl5 expression within in peribronchiolar regions (**Figure 4A**). Quantitative analysis targeted on the peribronchiolar regions confirmed significantly higher Ccl5 expression compared to controls (p = 0.002, q = 0.004, n = 5 one excluded control observation), while VEGF expression remained unchanged across experimental conditions (p = 0.29, q = 0.29, n = 5 one excluded control observation; **Figure 4B**).

**Figure 5:**
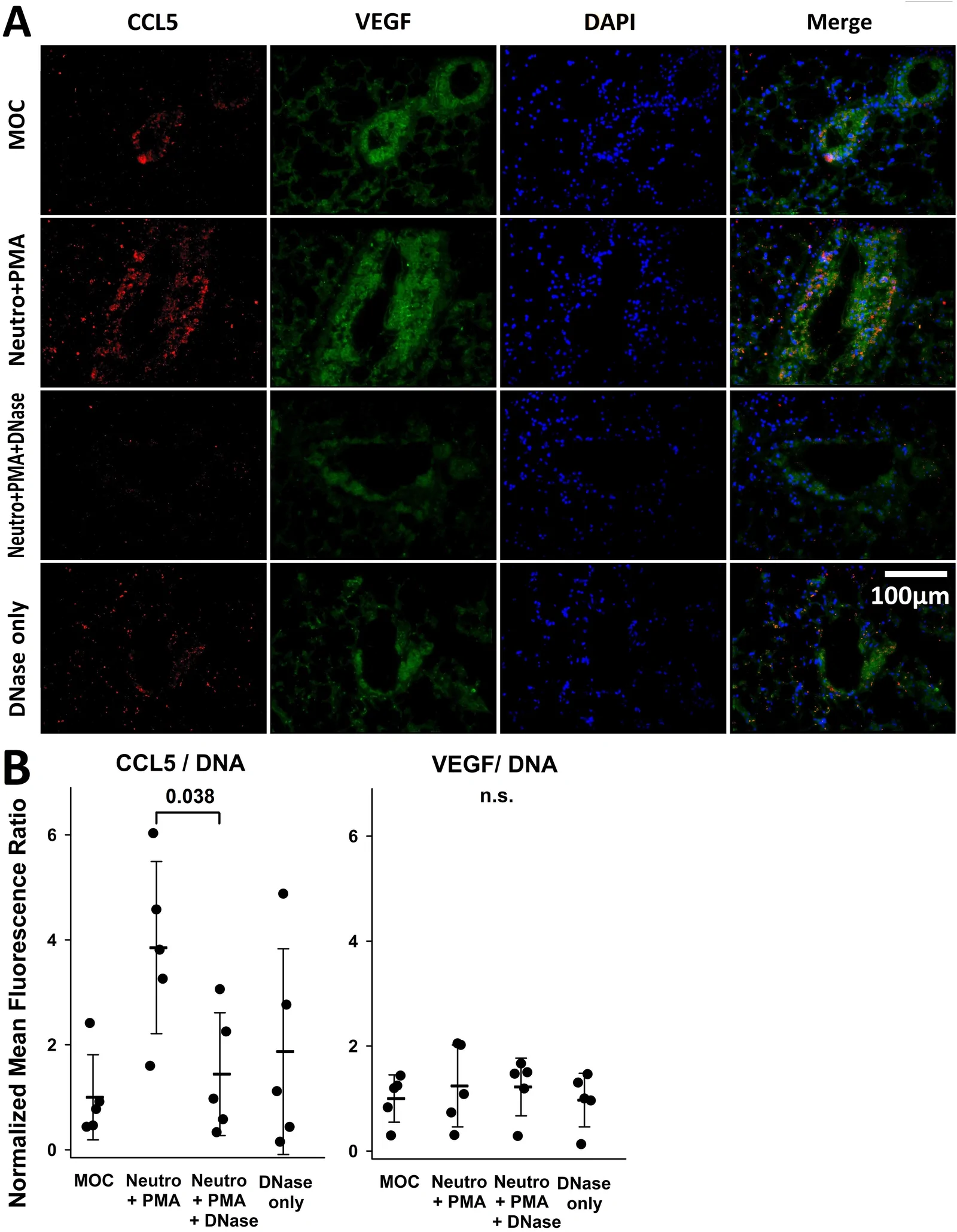
DNase1 attenuates NET-driven inflammatory activation in murine lung explants while preserving VEGF expression. **(A)** Immunofluorescence shows that DNase1 reverses the NET-induced increase in Ccl5, in peribronchiolar regions, indicating localised suppression of NET-mediated inflammatory signalling. **(B)** Automated quantitative image analysis confirms a significant reduction of Ccl5 intensity (normalised to total DAPI) compared with explants exposed to activated neutrophils (Neutro+PMA), whereas VEGF expression remains unchanged, suggesting that DNase1 mitigates inflammation without compromising angiogenic signalling by VEGF (n = 5 slices per condition from five animals, graphs show mean ± standard deviation, values above brackets indicate Holm-adjusted significance values). Ccl5, C-C motif chemokine ligand 5; CitH3, citrullinated histone H3; DAPI, 4′,6-Diamidin-2-phenylindol-dihydrochlorid,2-(4-Amidinophenyl)-6-indolcarbamidin; MOC, medium only control; PMA, phorbol 12-myristate 13-acetate; Neutro, neutrophils; Neutro+PMA, PMA-activated neutrophils; VEGF, vascular endothelial growth factor.

Addition of DNase1 to Neutro+PMA-exposed explants significantly reduced Ccl5 expression compared to Neutro+PMA alone (p = 0.019, q = 0.038, n = 5; **Figure 5**), restoring levels comparable to medium-only controls. VEGF expression was not significantly affected by DNase1 treatment under any experimental condition.

## Discussion

Using a tissue-architecture–preserving murine lung explant model, we show that exposure to activated human neutrophils produces robust *in situ* NET deposition, confirmed by colocalised citrullinated histone H3 (CitH3) and myeloperoxidase (MPO) signal, and triggers a coordinated chemokine response that is detectable within peribronchiolar regions of interest. DNase1 attenuates this inflammatory signal while leaving VEGF expression unchanged, indicating preserved VEGF expression alongside attenuation of NET-associated inflammation. These observations are relevant as motivation for ECMO support, in which neutrophil hyperactivation and NET formation are implicated in pulmonary morbidity, and for which NET-targeted pharmacological strategies remain insufficiently explored. The model further provides a tractable platform applicable to other neutrophilic airway pathologies in which NET-targeted therapy is under consideration, including ARDS, severe asthma, cystic fibrosis, and bronchopulmonary dysplasia.

In line with these findings, a comparable increase of Ccl5 in the serum was reported in a murine endotoxin-induced lung injury model (21). In the present data, quantitative immunofluorescence analysis confirmed that NET-associated induction of inflammatory markers was detectable within the tissue architecture itself. This expression was visually most compelling in the peribronchiolar regions and only sparsely observed in the distal parenchyma between bronchioles. Imaging fields were therefore centred on bronchioles for standardised data acquisition. This spatial pattern should be regarded as descriptive; it is consistent with localised peribronchiolar activation but does not constitute an unbiased peribronchiolar-versus-distal quantification. Taken together, the presence of activated neutrophils in proximity to murine lung tissue was associated with disseminated NET formation and localised activation of inflammatory signalling pathways in peribronchiolar regions. The finding that DNase1, which degrades extracellular DNA, markedly attenuated the induced inflammatory response is consistent with a NET-dependent component of the observed inflammation. However, because DNase1 degrades extracellular DNA irrespective of its cellular source, this single nuclease-based manipulation cannot by itself establish specificity for extracellular traps from neutrophils. A further consideration is the cellular source of the CCL5 signal: because the anti-CCL5 antibody detects both human and murine CCL5, it does not provide attribution to the murine lung tissue under exposure.

Further translational studies are required to assess the applicability of these findings in human systems. Safety and efficacy of anti-NET strategies as an adjunct to ECMO therapy warrant careful evaluation. NET formation has been associated with immunothrombosis within extracorporeal circuits, where extracellular DNA and histones may contribute to oxygenator clot formation and circuit dysfunction (22). Enzymatic degradation of NET structures by DNase1 could theoretically reduce such thrombotic deposition and improve circuit stability. Moreover, NET-driven microvascular thrombosis within the pulmonary circulation may aggravate lung injury during extracorporeal support (7). Targeted modulation of NET burden might therefore help limit both circuit-related thrombo-inflammatory responses and pulmonary immunothrombosis. However, the clinical relevance of these mechanisms during ECMO remains to be determined in *in vivo* and human studies.

Our findings extend recent biomaterial-focused investigations of ECMO-associated NET biology. Foltan et al. demonstrated that granulocytes adhere uniformly to commercially available polymethylpentene gas-fibre membranes irrespective of surface coating, yet the proportion of neutrophils undergoing NET formation differs substantially between antithrombogenic coatings, identifying material-driven NET formation as one upstream trigger of intra-circuit thrombogenesis (5). Our model addresses the complementary question—what tissue-level inflammatory programmes do circuit-derived NETs elicit within the lung parenchyma? Translational support comes from the prospective cohort by Haus and colleagues, in whom elevated NET-precursor levels in ECMO-patient blood tracked with the development of thrombocytopenia (23). Together, these data delineate a coherent material-to-tissue axis—from coating-dependent NET formation at the artificial surface to peribronchiolar chemokine amplification within the lung parenchyma—that has not previously been resolved within a single experimental framework.

In an *in vitro* membrane oxygenation circuit, Winnersbach and colleagues observed that platelet depletion reduces NET formation but does not prevent clotting (20), implying that platelet-targeted strategies alone are insufficient to disrupt circuit-related immunothrombosis. Our data argue for a complementary approach in which the extracellular DNA scaffold itself is enzymatically removed—an intervention that, on the basis of preclinical work in intestinal ischaemia–reperfusion injury (13), testicular torsion (14), and host-DNase-deficient venous occlusion models (15), can be achieved without amplifying haemorrhagic risk. This is mechanistically attractive in the ECMO context, where conventional anticoagulation must be carefully titrated against bleeding complications that already account for the leading cause of morbidity particularly in neonatal recipients (1–3).

It is important to distinguish the present pharmacological strategy from approaches that abolish NET formation altogether. Eghbalzadeh et al. showed that PAD4-deficient mice exhibit paradoxically aggravated inflammation after myocardial infarction, suggesting that prevention of histone citrullination can disrupt homeostatic anti-inflammatory functions of NETs (10). DNase1, in contrast, selectively degrades extracellular chromatin scaffolds only after their release, leaving upstream PAD4-dependent signalling intact—a mechanistic distinction that may be desirable when intervening in patients for whom complete NET ablation is undesirable. The preserved VEGF expression in our DNase1-treated explants is also consistent with the work of Sun and colleagues, who reported that heparin improves alveolarization and vascular development in hyperoxia-induced bronchopulmonary dysplasia by inhibiting NET formation (24). The concurrent attenuation of NET-associated CCL5 induction and preserved VEGF expression provides mechanistic plausibility for adjunctive NET-targeted therapy in neonatal lung-injury syndromes in which preservation of the angiogenic programme is essential for alveolar–capillary repair. NETs themselves exert context-dependent effects on angiogenesis, promoting neovascularisation in some settings (8, 9) while impairing vascular repair in others (11); this dual role reinforces the importance of confirming that extracellular-DNA-directed intervention does not compromise VEGF-dependent signalling.

## Limitations

Several limitations require transparent acknowledgement. The ex vivo lung explant model does not reproduce the haemodynamic forces, intravascular cellular trafficking, coagulation cascade activation, or neurohumoral signalling that characterise *in vivo* ECMO physiology. The absence of circulating platelets precludes assessment of NET-mediated thrombogenic consequences – arguably the most clinically critical dimension of NET biology in ECMO patients. Future studies employing ex vivo perfused lung preparations or large-animal ECMO models with continuous circuit monitoring are essential for evaluating the antithrombotic potential of DNase1 under conditions that more closely approximate the clinical setting.

A further constraint of the present study is the use of PMA as a pharmacological NET inducer. PMA elicits a robust but non-physiological NETotic response that does not recapitulate the shear-, contact-, and complement-mediated triggers operative within an ECMO circuit; future studies should therefore corroborate these observations with biomaterial-, hypoxia-, or platelet-driven NET stimulation under flow conditions. Hence, this study was not designed to assess the thrombogenic consequences of NETs formation. Given the well-established contribution of NETs to immunothrombosis (7, 25), particularly in extracorporeal circulation systems (5, 22), this would be an important objective for future investigations. An additional limitation is the interspecies design, as human neutrophils were combined with murine lung explants. While the use of human neutrophils enhances translational relevance by reflecting clinically pertinent NET behaviour, cross-species interactions may not fully reproduce species-specific inflammatory signalling. Our findings should therefore be interpreted with caution and validated in homologous human or *in vivo* models. Moreover, the lung explants and the donor neutrophils were obtained from adult tissue, whereas part of the clinical motivation relates to neonatal ECMO; developmental differences in neutrophil function and pulmonary immunity mean that the present model does not directly recapitulate the neonatal context.

Finally, VEGF was assessed as a marker of angiogenic signalling (24), however, it represents only one component of a complex regulatory network governing neoangiogenesis.

## Conclusion

Exposure of intact murine pulmonary tissue to activated human neutrophils is associated with localised inflammatory amplification, characterised by peribronchiolar Ccl5 upregulation, that is attenuated by DNase1 without a detectable change in VEGF expression. These findings provide a hypothesis-generating framework for NET-associated pulmonary inflammation that is attenuated by extracellular DNA degradation and provide a rationale for further investigation of DNase1 as a candidate adjunct anti-inflammatory strategy in (neonatal) ECMO. The preserved VEGF expression alongside attenuation of NET-associated inflammation is compatible with selective NET targeting as a potential complement to conventional anticoagulation; however, the present static, flow-free model includes no thrombotic endpoint and therefore cannot speak to immunothrombosis or to a reduction in haemorrhagic risk. The model is additionally applicable to other neutrophilic airway pathologies in which NET-targeted therapy is under consideration, including ARDS, severe asthma, cystic fibrosis, and bronchopulmonary dysplasia.

## Data Availability

The data sets presented in this study can be found online. https://doi.org/10.11588/DATA/IOAMVQ.
